# TMEM105 modulates disulfidptosis and tumor growth in pancreatic cancer via the β-catenin-c-MYC-GLUT1 axis

**DOI:** 10.7150/ijbs.104598

**Published:** 2025-02-18

**Authors:** Yifan Yin, Yixuan Sun, Hongfei Yao, Feng Yu, Qinyuan Jia, Chengyu Hu, Yuheng Zhu, Zonghao Duan, Dejun Liu, Yongwei Sun, Yanmiao Huo, Minwei Yang, Wei Liu

**Affiliations:** 1 Department of Biliary-Pancreatic Surgery, Ren Ji Hospital, School of Medicine, Shanghai Jiao Tong University, Shanghai, 200127, China.; 2 Department of Gynecology and Obstetrics, Shanghai Sixth People's Hospital Affiliated to Shanghai Jiao Tong University School of Medicine, Shanghai, 200235, China.; 3 Department of General Surgery, Pancreatobiliary Surgery Center, Huadong Hospital Affiliated to Fudan University, Shanghai, 200040, China.

## Abstract

**Background:** Pancreatic cancer (PCa) is one of the most malignant diseases in the world. Different from ferroptosis and apoptosis, disulfidptosis is a novel type of cell death. The role of disulfidptosis in PCa remains uncovered.

**Methods:** Disulfidptosis-related lncRNAs were identified based on TCGA-PAAD database. The disulfidptosis-related predict signature was constructed and verified by bioinformatic analysis. TCGA and GTEx database and Renji tissue microarray (TMA) were applied to determine *TMEM105* and its clinical significance. F-actin and PI staining were performed to detect disulfidptosis of PCa cells. The biological function of *TMEM105* was investigated by loss-of-function and gain-of-function assays. RNA pull-down and LC-MS/MS analysis were employed to detect *TMEM105* interacted proteins. The tissue samples from PCa patients with PET-CT information were utilized to validate the *TMEM105*-β-catenin-c-MYC-GLUT1 pathway in clinical settings.

**Results:** A disulfidptosis-related predict signature, which was comprised of six lncRNAs, was constructed and validated by bioinformatic analysis. *TMEM105* was identified as disulfidptosis-related lncRNA whose high expression predicted a poor prognosis in PCa. Functional studies revealed that *TMEM105* promoted the growth and mitigated the disulfidptosis in PCa. Mechanically, *TMEM105* upregulated the expression of β-catenin by maintaining the protein stability through the proteosome pathway. The forced expressed β-catenin increased the expression of glycolysis-related transcription factor c-MYC, thus induced the transcription activity of GLUT1.

**Conclusion:** These results revealed the growth acceleration and the disulfidptosis mitigation function of *TMEM105* in PCa. Targeting the *TMEM105*-β-catenin-c-MYC-GLUT1 pathway could be a potent therapy for PCa patients.

## Introduction

Globally, the morbidity of pancreatic cancer (PCa) ranks the fourth among all cancer types 1. PCa is characterized by high malignancy, invasiveness and less than 7% of five-year survival rate 2. Pancreatic ductal adenocarcinoma (PDAC) is the most prevalent type of PCa, accounting for approximately 80% of all cases 3. Clinically, surgery is the primary treatment for pancreatic cancer. However, only a small number of PCa patients are eligible for surgery while fewer patients could achieve long-term survival after surgical treatment 4, 5. Therefore, exploring the mechanisms of PCa tumorigenesis and developing effective treatment strategies are urgently needed.

Resistance toward regulated cell death is a typical characteristic of cancer 6. It refers to upregulation of cancer cell growth and failure of therapy response 7. For instance, defects of apoptosis could lead to drug resistance and metastasis of cancer cells 8. Disulfidptosis, different from known types of regulated cell death, is a novel type of cell death. Solute carrier family 7 member 11 (SLC7A11) is a critical transporter protein, playing a key role in mediating cellular cystine uptake. Importantly, cystine serves as a crucial element for synthesizing glutathione and regulating cellular oxidative stress responses. However, as two-edged sword, cystine is cytotoxic as well. Normally, disulfidptosis was triggered under the circumstance where glucose was deprived in high-SLC7A11-expressed cancer cells. In this case, GLUT1 inhibitor treatment could induce depletion of NADPH in the pentose phosphate pathway (PPP), causing disulfide stress in cytoskeleton and the collapse of structural proteins, ultimately leading to disulfidptosis 9. Nevertheless, the correlation of pancreatic cancer and disulfidptosis has not been fully elaborated.

Long non-coding RNAs (lncRNAs) refer to transcripts with over 200 nucleotides which couldn't be translated into proteins. It could regulate RNAs, DNAs and proteins to influence the cancer cells differentiation, proliferation and migration 10-12. Numerous studies have verified that lncRNAs could serve as biomarker or therapeutic target in PCa. It was reported that the lncRNA *TMEM105* played an important role in promoting breast cancer growth and metastasis by regulating tumor glycolysis 13. In the research of thyroid papillary carcinoma, *TMEM105* was reported to function as a cell cycle-related lncRNA in predicting progression-free survival time 14. Besides, it was also a ferroptosis- and immune-related lncRNA for predicting patient prognosis in breast infiltrating duct and lobular carcinoma 15. In addition, *TMEM105* was believed to play a crucial role in coronary artery disease 16. Anyhow, its function in PCa and disulfidptosis remained unclear.

In this study, *TMEM105* was identified as a disulfidptosis-related biomarker to predict the prognosis of PCa patients, whose expression was significantly increased in tumorous tissues compared with normal tissues. Mechanically, *TMEM105* engages a β-catenin-c-MYC-GLUT1 cascade to promote tumor growth and antagonize disulfidptosis in PCa.

## Method and materials

### Date collection

178 PCa patients with corresponding clinical data were obtained from TCGA (https://portal.gdc.cancer.gov/repository).

### Screening of disufidptosis‑related lncRNAs

*GYS1*, *LRPPRC*, *NCKAP1*, *NDUFA11*, *NDUFS1*, *NUBPL*, *OXSM*, *RPN1*, *SLC3A2* and *SLC7A11* were defined as disulfidptosis-related genes from previous research 17. Gene annotation was utilized for the identification and screening of lncRNAs. After Pearson correlation analysis (|Pearson R|>0.35, P value<0.001), 19 lncRNAs were selected. The “ggplot2”, “dplyr”, “limma” and “ggalluvial” packages were used to visualize the Sankey diagram of disulfidptosis-related genes and disulfidptosis-related lncRNAs.

### Establishing and verifying the risk signature

Randomly, pancreatic cancer patients were divided into training and testing two groups by 1:1. The univariate Cox regression analysis was used on the co-expressed lncRNAs to select the prognostic-related lncRNAs of the training group (p<0.05). Then a least absolute shrinkage and selection operator regression analysis (LASSO) coupled with multivariate Cox regression analysis were performed on the establishment of the risk signature. The risk score was calculated by the following formula:

Risk score = 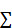
[Exp (lncRNA)×coef (lncRNA)]

The Exp(lncRNA) indicated the lncRNAs expression and the coef(lncRNAs) indicated the corresponding coefficient. The receiver operating characteristic curves (ROC) and the area under the time-dependent ROC curves (AUCs) was constructed by “survmine” and “survival” packages. Finally, the testing cohort and entire cohort was applied to validate the risk signature.

### Principal component analysis (PCA)

PCA is performed as a statistical method to reduce dimensionality and visualize the 19 disulfidptosis-related genes, 6 disulfidptosis-related lncRNAs, the risk signature and whole-genome expression profiles. The “Scatterplot3d” package was applied for the PCA visualization.

### Patients and tissue microarray (TMA) construction

In this article, there are two groups of samples. The first group: 149 PCa and paired normal tissues from patients receiving surgical treatment in our institution were included to construct TMA. The second group:14 cases of PCa patients and their corresponding PET-CT information. The information of pathology was obtained from the Pathology Department. The overall survival time was calculated from date of receiving surgical treatment to the PCa-related death (approval number: RA-2024-196).

Two pathologists were invited to independently score the Tissue Microarray (TMA). The scoring criteria for positive staining area were as follows: 0%-5% scored as 0, 6%-35% scored as 1, 36%-70% scored as 2, and above 70% scored as 3. Staining intensity was graded with no staining as 0, weak staining as 1, moderate staining as 2, and strong staining as 3. The final score was calculated by multiplying the score for positive staining area by the staining intensity score. A score of 0 was denoted as “-”, 1-3 as “+”, 4-6 as “++", and above 6 as "+++”. When distinguishing between high and low expression groups, a final score below 4 was classified as *TMEM105* low expression group, and a score above 4 was classified as the *TMEM105* high expression group.

### Cell culture and reagents

Pancreatic cell lines (HPNE, Capan-1, Mia CaPa-2, Patu8988, PANC-1 and SW-1990) were obtained from Cell Bank of the Chinese Academy of Sciences (Shanghai, China). Cells were all cultured in the medium instructed by American Type Culture Collection (ATCC) protocols. The reagents used in the study were listed as follows: dithiothreitol (DTT) (MCE, HY-15917), BAY-876 (MCE, HY-100017), SKL2001 (MCE, HY-101085).

### Subcellular fractionation

Cytoplasmic and nuclear RNA was separated by using commercial kit (Norgen Biotek, Canada) according to instructions. Briefly, 400 μL of lysis buffer was added to the cell pellet, followed by incubation on ice for 10 minutes and centrifugation at 14,000 rpm for 15 minutes. Hence, the cytoplasmic components were obtained in supernatant while the nuclear was in pellet. Subsequently, the cytoplasmic and nuclear samples were dissolved and mixed with 800μL of 1.6M sucrose solution and centrifuged at 14,000 rpm for 15 minutes at 4°C to purify the cytoplasmic and nuclear fractions. Finally, the purified RNA was dissolved in Trizol.

### Knockdown and overexpression assays

The small interfering RNAs (siRNAs) were purchased from Gene Pharma (Shanghai, China). Cells were seeded at an appropriate density in a 6-well plate overnight. siRNA (50 pmol) or 2.5 µl of Dharmacon transfection reagent were added separately into 200 µl of Opti-MEM medium and incubate for 5 minutes. Then, the two solutions were mixed, incubated for 20 minutes, and added to the 6-well plate for 48 to 72 hours. The sequence was as si-*TMEM105*-1: 5'-CCCAUAGCUGACACUUCUA-3' (sense), 5'-UAGAAGUGUCAGCUAUGGG-3' (antisense); si-*TMEM105*-2: 5'-GGCAAGCUCUGAUCUUACA-3' (sense), 5'-UGUAAGAUCAGAGCUUGCC-3' (antisense). Sequence of sh-*TMEM105*-1 and sh-*TMEM105*-2 was same as that of siRNAs.

The *TMEM105* overexpression was achieved using Ubi-MCS-SV40-Puromycin synthesized by Genechem (Shanghai, China). Stable cell line was selected and maintained in the complete medium with 5μg/mL of puromycin.

### Quantitative real‑time PCR (qPCR)

qPCR was performed as described previously 18. Primer sequences are listed as follows: *TMEM105* forward, 5'- ATGAAGATAAGAAGGCGA-3', reverse, 5'- GGTGAAAAACACGATGAG-3'; *β-catenin* forward, 5'-CACAAGCAGAGTGCTGAAGGTG-3', reverse, 5'- GATTCCTGAGAGTCCAAAGACAG-3'; *c-MYC* forward, 5'-GCCTCAGAGTGCATCGAC-3', reverse, 5'-TCCACAGAAACAACATCG-3'; *GLUT1* forward, 5'-ATTGGCTCCGGTATCGTCAAC-3', reverse, 5'-GCTCAGATAGGACATCCAGGGTA-3'; human *18s* forward, 5'- GGCCCTGTAATTGGAATGAGTC-3', reverse, 5'- CCAAGATCCAACTACGAGCTT-3'; Relative mRNA expression levels were calculated using the 2^-ΔΔCt^ method and normalized to 18s RNA.

### Western blotting (WB)

WB was performed as described previously. 19 Anti-β-catenin (Proteintech, Cat. No. 51067-2-AP), anti-GLUT1 (Proteintech, Cat. No. 21829-1-AP), anti-c-MYC (Proteintech, Cat. No. 10828-1-AP) and anti-β-Actin (Abcam, Cat. No. ab8226) were applied.

### Proliferation and migration assays

For CCK-8 assays, cells were seeded at a density of 1,500 cells per well in a 96-well plate with triplicate technical replication per group. The cells were then incubated in a cell culture incubator at 37°C with 5% CO2 for 5 days. On each day, CCK-8 was added and incubated for 1.5 hours. Finally, cell viability was measured under the absorbance wavelength at 450 nm.

For colony formation assays, cells were seeded at a density of 1500 cells per well in a 6-well plate. After an incubation period of 7-10 days, the supernatant was discarded, the cells were washed with PBS, fixed with 4% paraformaldehyde, and stained with crystal violet. Subsequently, images were obtained using a microscope.

For transwell assays, 600μl complete culture medium was added to the lower chamber. After resuspended in serum-free medium at a density of 6 × 10^4^ cells per well, cells were added to the upper chamber of transwell. Following a 12-hour incubation, the cells were fixed with 4% paraformaldehyde and stained with crystal violet. The chamber was imaged under the microscope.

### Subcutaneous xenograft models and patient derived xenograft (PDX) models

6-8 weeks male athymic nu/nu mice were applied for subcutaneous injection of Mia CaPa-2 cells with stable *TMEM105* knockdown, Patu8988 cells with stable *TMEM105* overexpression, along with control cells, at a cell concentration of 2 × 10^6^ cells/100 μl. Tumor volume was measured every four days using a caliper. Tumor volume was calculated as (long diameter × short diameter × short diameter × 1/2). After one month, the mice were euthanized by cervical dislocation under anesthesia, and the tumors were excised for analysis. PDX models were constructed as described previously 20.

### EdU detection, cytoskeleton staining and Immunofluorescence (IF) staining

For the EdU detection, cells were cultured in an 8-well chamber slide (ibidi, Cat. No. 80826) at appropriate density. After the cells attached to the chamber, 5-Ethynyl-2'-deoxyuridine (EdU) assay commercial kit (ShareBio, Cat. No. SB-C6015) was applied according to manufacturer's instructions. For the cytoskeleton staining, cells were cultured in an 8-well chamber slide (ibidi, Cat. No. 80826) at appropriate density overnight. 4% paraformaldehyde were added into the chamber at room chamber for 15 minutes. Subsequently, samples were treated with 0.4% Triton X-100 for 5 minutes and stained with 594-phalloidin (ShareBio, Cat. No. SB-YP0052). IF staining was conducted as described previously. Anti-GLUT1 (Proteintech, Cat. No. 21829-1-AP) was used as primary antibody in IF staining assay 20. All samples were stained with DAPI and visualized by confocal microscope (Zeiss, Germany).

### Flow cytometry

Cells were seeded in 12-well plates at the density of 5 × 10^5^ and cultured overnight. Then the cells were cultured in glucose medium without DTT, in glucose-free medium without DTT and in glucose-free medium with DTT (0.25mmol/L) for about 12 h. PI (ShareBio, Cat. No. SB-Y6002) was used to stain the dead cells and the dead cells was measured by flow cytometry.

### Glucose consumption assays

Cells were seeded and treated in the 6-well plate for 24 hours before being collected for the following experiment. Glucose assay kit (Invitrogen, Cat. A22189) was applied for the glucose consumption assay. All procedures were following the manufacturer's protocol. The enzyme working solution from the kit was added to each sample, followed by incubating at 37°C for 10 minutes. The absorbance A of the samples was measure at 505 nm of wavelength. The glucose consumption =5.55 × (A complete DMEM - A blank) / (A standard - A blank) - 5.55 × (A treatment - A blank) / (A standard - A blank). The relative glucose consumption was standardized by the number of cells.

### NADP^+^/NADPH measurements

The intracellular levels of NADP^+^/NADPH were conducted according to the instructions of the commercial kit (S0179, Beyotime, China). Cells were seeded into 6-well plates at the proper density and subjected to the prescribed treatments. The cells were lysed using 200 μl of lysis buffer, and the samples were divided into two parts. Sample A was heated at 60°C for 30 minutes to allow NADP^+^ convert to NADPH, while sample B was kept on ice. Subsequently, the working buffer was added, and the samples were incubated for 10 minutes while avoiding light exposure. Finally, spectroscopic measurements were performed at 450 nm. The NADP^+^/NADPH ratio = (intensity of sample A)/ (intensity of sample B - intensity of sample A).

### RNA pull-down assays and LC-MS/MS analysis

The resultant plasmid DNA was linearized with restriction enzyme NotI. Biotin-labeled RNAs were *in vitro* transcribed with the Biotin RNA Labeling Mix (Roche) and T7 RNA polymerase (Roche), treated with RNase-free DNase I (Roche) and purified with the RNeasy Mini Kit (Qiagen). Cells extract was mixed with biotinylated RNA (2 μg/100 pmol). Washed streptavidin agarose beads (100 ml, Invitrogen) were added to each binding reaction and further incubated at room temperature for 0.5 h. Beads were washed 5 times and boiled in SDS buffer. Finally, the retrieved protein was determined by LC-MS/MS and western blot analysis. For LC-MS/MS analysis, proteins were resolved by SDS-PAGE and total protein band was excised, eluted and digested by trypsin. Digests were analyzed by tims TOF pro2 system (Bruker). MS data was analyzed by PeakStudio 11 (Bioinformatics Solutions) by searching the Uniprot protein database.

### Immunohistochemistry (IHC), *in situ* hybridization (ISH) and TUNEL assay

The experimental methods were performed as described previously 21. The following probes or antibodies were used: *TMEM105* specific probe synthesized by servicebio (Wuhan, China), anti-Ki-67 (1:1000, Cat. No. ab15580 Abcam), anti-SLC7A11 (Proteintech, Cat. No. 26864-1-AP), anti-c-MYC (Proteintech, Cat. No. 10828-1-AP), anti-GLUT1 (Proteintech, Cat. No. 21829-1-AP), TUNEL commercial kits (Absin, Cat. No. abs50022).

### Statistical analysis

The data were presented as the mean values ± SD. Statistical analyses were performed via GraphPad Prism software 8. Two-tailed Student's t-tests were used for two group comparisons, and one-way analysis of variance (ANOVA) with a Tukey post-hoc test was used for multiple comparisons. Cell viability and tumor volume were analyzed by two-way ANOVA.

## Results

### Identifying disulfidptosis-related lncRNAs, construction and validation of the disulfidptosis-related predictive signature in PCa

**Figure 1:**
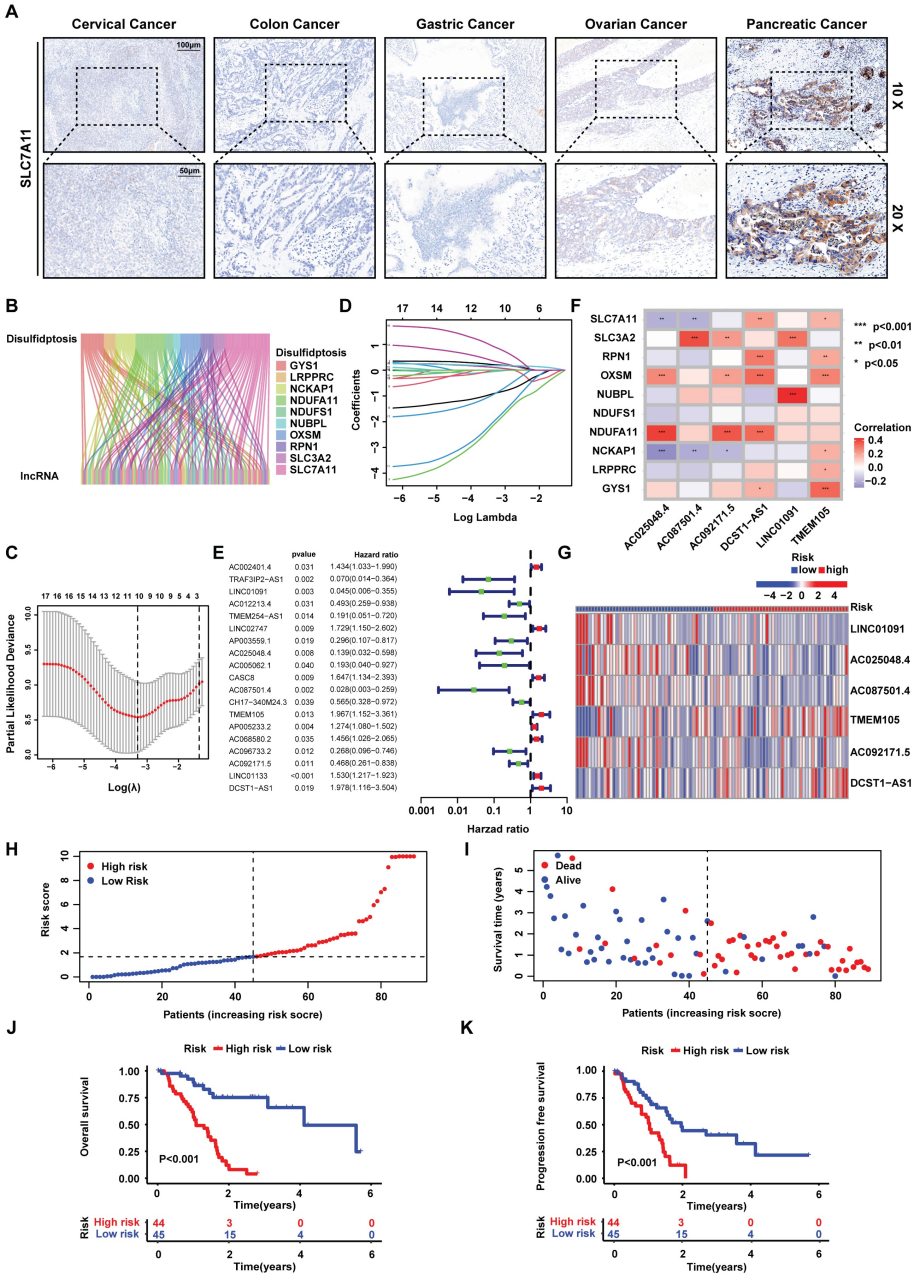
** Identifying disulfidptosis-related lncRNAs, construction and validation of the disulfidptosis-related predictive signature in PCa. (A)** SLC7A11 expression in five cancer types analyzed by IHC staining. Scale bar: 10x (100 μm) and 20x (50 μm). **(B)** Sankey diagram of disulfidptosis-related genes and disulfidptosis-related lncRNAs. **(C)** Cross-validation plot for the penalty term. **(D)** LASSO expression coefficient plot of disulfidptosis-related lncRNAs. **(E)** Forest plot of univariate COX regression analysis results for disulfidptosis-related lncRNAs. **(F)** Heatmap of the correlation between disulfidptosis-related lncRNAs and disulfidptosis-related genes. **(G)** Risk heatmap of the training set. **(H)** Distribution plot of risk scores in the training set. **(I)** Scatter plot of survival status in the training set. **(J)** Kaplan-Meier (KM) analysis for overall survival (OS) in the training set based on the TCGA database. **(K)** KM analysis for PFS in the training set based on the TCGA database.

Disulfidptosis is a novel type of cell death, which is triggered under the circumstance where glucose is deprived in high-SLC7A11-expressed cancer cells. Therefore, IHC staining was used to detect the SLC7A11 expression of 5 common cancer types. Obviously, PCa expressed higher level of SLC7A11 than others (Figure 1A). To reveal the role of disulfidptosis in PCa and predict the overall survival (OS) of PCa patients, we constructed a risk signature based on disulfidptosis-related lncRNAs by bioinformatic analysis. Firstly, the RNA sequence profile of PCa samples and corresponding clinicopathological information was obtained from TCGA. In the meantime, 10 disulfidptosis -related genes (*GYS1*, *LRPPRC*, *NCKAP1*, *NDUFA11*, *NDUFS1*, *NUBPL*, *OXSM*, *RPN1*, *SLC3A2* and *SLC7A11*) were selected based on the previous reports 17. In this situation, we acquired 100 disulfidptosis-related lncRNA (|Pearson R|>0.35, and p<0.001) (Figure 1B).

On the side, a total of 178 PCa patients were divided into training and testing groups. Univariate Cox regression was used to screen prognostic related lncRNAs in training cohort. Consequently, 19 lncRNAs (*AC002401.4*, *TRAF3IP2-AS1*, *LINC01091*, *AC012213.4*, *TMEM254-AS1*, *LINC02747*, *AP003559.1*, *AC025048.4*, *AC005062.1*, *CASC8*, *AC087501.4*, *CH17-340M24.3*, *TMEM105*, *AP005233.2*, *AC068580.2*, *AC096733.2*, *AC092171.5*, *LINC01133* and *DCST1-AS1*) were achieved to construct risk signature by LASSO regression analysis (Figure 1C-E). Afterwards, six lncRNAs which (*AC025048.4*, *AC087501.4*, *AC092171.5*, *DCST1-AS1*, *LINC01091* and *TMEM105*) affecting the prognosis independently were acquired by multivariate Cox regression, correlating with disulfidptosis-related genes closely (Pearson test) (Figure 1F). Inside, *LINC01091*, *AC025048.4*, *AC087501.4*, *AC092171.5* and *DCST1-AS1* were protective factors while *TMEM105* was the only poor prognostic factors in the risk signature. Thus, a risk signature based on six disulfidptosis-related lncRNAs was then developed to predict the patients OS. The risk scores for all samples were calculated as the following formula: risk score = -3.7619 ×Exp (*LINC01091*) + -1.9756 ×Exp (*AC025048.4*) + -3.3182 ×Exp (*AC087501.4*) + 0.9454 ×Exp (*TMEM105*) + -1.8259 ×Exp (*AC092171.5*) + 1.8009 ×Exp (*DCST1-AS1*).

The training group was divided into high- and low- risk groups according to the median risk score. The correlation between the expression of 6 lncRNAs and every sample was depicted by the heatmap (Figure 1G). Moreover, the risk score was negatively correlated with the survival state (Figure 1H-I), while high-risk group presented worse OS and PFS compare to low-risk group (Figure 1J-K). Subsequently, through the univariate and multivariate Cox regression analysis, it was found that the risk signature could be deemed as independent risk factor to influence OS (P<0.001). To confirm the feasibility of risk signature, we checked it in testing group and entire cohort to reach the similar conclusion (Figure S1-6). We performed the PCA analysis of all genes, 10 disulfidptosis-related genes, 19 disulfidptosis-related lncRNAs and the risk signature (Figure S7). It was obvious that the risk signature could divide the samples into 2 groups perfectly.

### High expression of TMEM105 predicts the poor prognosis in PCa

**Figure 2:**
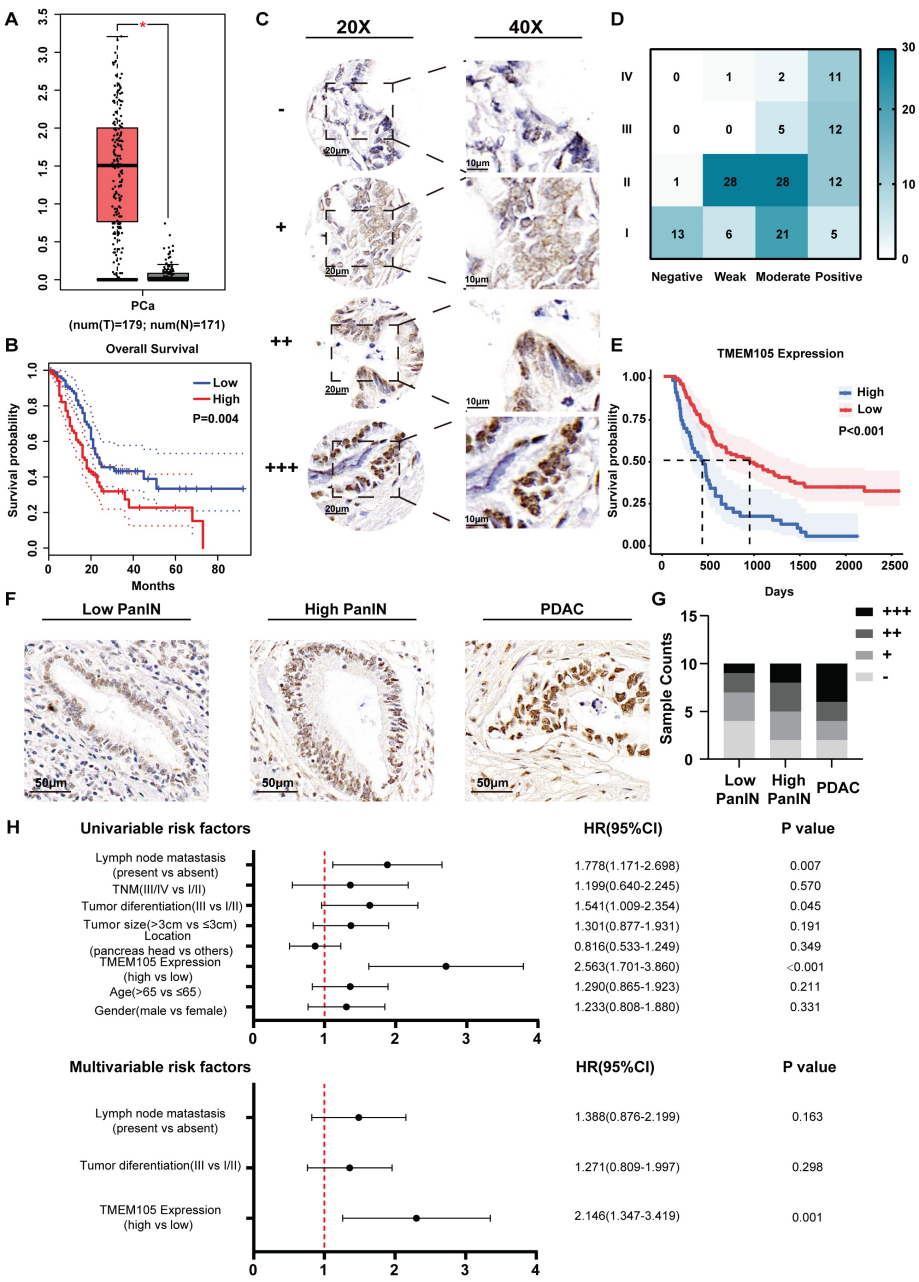
** High expression of *TMEM105* predicts a poor prognosis in PCa. (A)** Expression profile of *TMEM105* in TCGA database. **(B)** KM analysis of OS in patients with high or low *TMEM105* expression by GEPIA. **(C)** Standard ISH grading images of *TMEM105* expression in 149 pancreatic cancer tumors. Scale bar: 20x (20 μm) and 40x (10 μm) for negative (-), weak (+), moderate (++), and strong (+++) expressions. **(D)** Heatmap of correlation between *TMEM105* expression and TNM stage based on ISH grading. **(E)** The KM analysis for the correlation between OS rate and *TMEM105* expression based on the Renji cohort. **(F)** Representative standard ISH staining images of various stages of PCa progression in the Renji cohort, including PanIN (pancreatic intraepithelial neoplasia) and PDAC (pancreatic ductal adenocarcinoma); (scale bar: 50 μm). **(G)** Statistical analysis for ISH staining in different stages of PDAC, the grades were classified as negative (-), weakly positive (+), moderately positive (++), and strongly positive (+++). **(H)** Univariate and multivariate Cox regression analyses of clinical and pathological factors in the Renji cohort.

To further investigate the role of disulfidptosis-related lncRNAs in PCa, we initially conducted Gene Expression Profiling Interactive Analysis (GEPIA) 22. It was found that there was significantly higher expression level of *TMEM105* in PCa tumor tissues compared to adjacent normal tissues (Figure 2A). In consistant with the pro-tumoral effect predicted in the risk signature, high expression of *TMEM105* also predicted a poor prognosis (Figure 2B). However, protective factors (*LINC01091*, *AC025048.4*, *AC087501.4*, *AC092171.5*) presented in the risk signature showed no significant differences in term of expression or prognosis in PCa. Consequently, *TMEM105* was identified as the critical gene in this study to detect *TMEM105* expression, 149 PCa as cases and their adjacent tissues in TMA were examined and scored (Figure 2C). It was found that *TMEM105* was predominantly located in the nucleus of pancreatic cancer cells. Plus,* TMEM105* showed higher expression in advanced stages of PCa compared to lower stage and correlated with poor prognosis (Figure 2D-E). Then, indicated number of ISH images was selected for statistical analysis (Figure 2F). As a result, the expression of *TMEM105* was positively correlated with the pathological grade of PCa (Figure 2G). Subsequently, PCa samples in the Renji TMA were assigned into high- and low- expression groups based on *TMEM105* expression. In the meantime, various clinical indicators were taken into consideration. Consequently, lymph node metastasis, tumor differentiation, and *TMEM105* expression were identified to impact the prognosis of pancreatic cancer significantly by univariate COX regression analysis. Besides, multivariate COX regression analysis was also conducted while *TMEM105* expression was confirmed as an independent risk factor influencing the prognosis of PCa (Figure 2H).

### TMEM105 promotes PCa cells growth *in vitro* and *in vivo*

**Figure 3:**
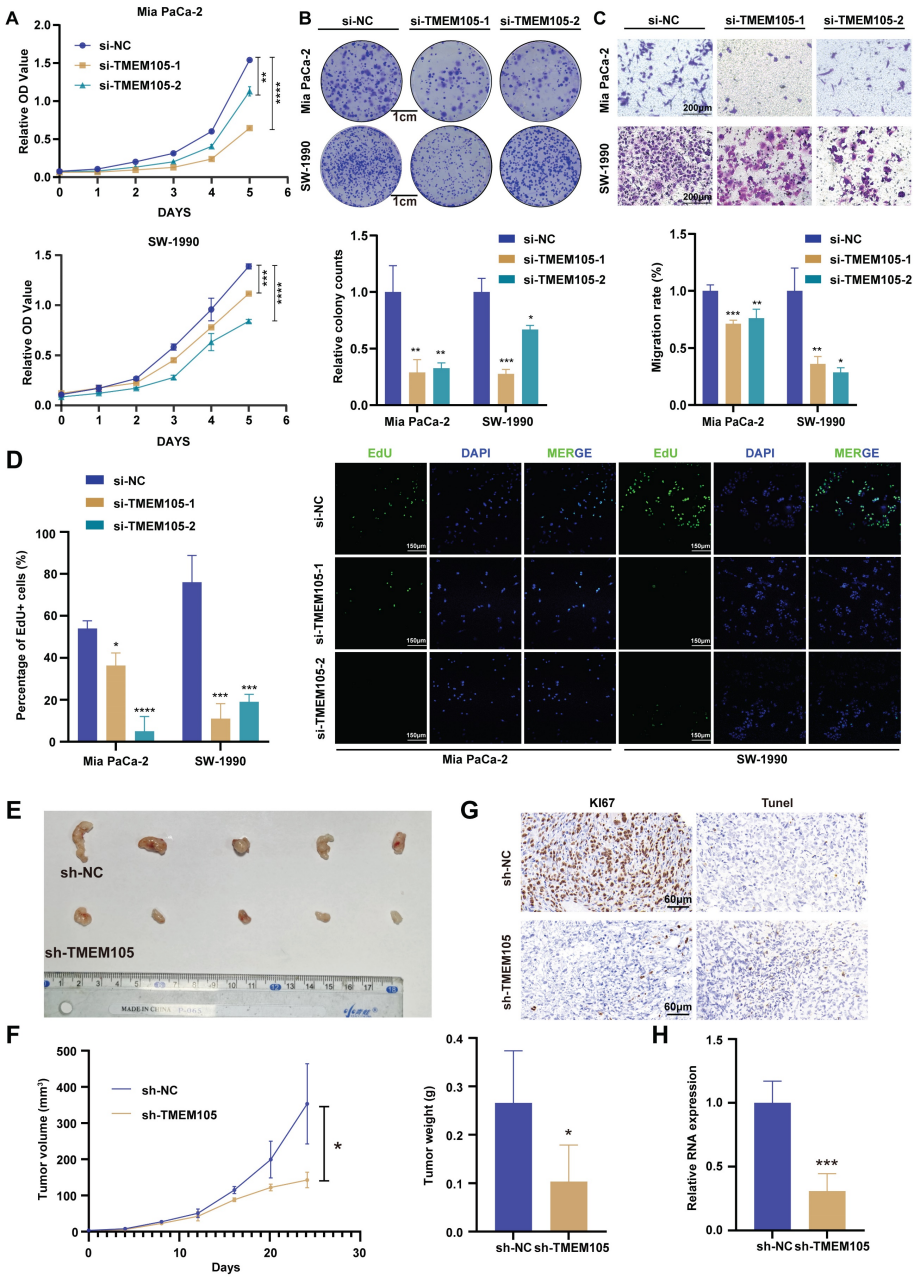
***TMEM105* promotes the growth of PCa both *in vitro* and *in vivo*. (A)** Cell viability of *TMEM105* knockdown Mia PaCa-2 and SW-1990 cells. **(B)** The colony formation assays of *TMEM105* knockdown Mia PaCa-2 and SW-1990 cells and its analysis (scale bar: 1 cm). **(C)** Cell migration assay of *TMEM105* knockdown Mia PaCa-2 and SW-1990 cells and its analysis (scale bar: 200 μm). **(D)** EdU staining of *TMEM105* knockdown Mia PaCa-2 and SW-1990 cells and its analysis (scale bar: 150μm). **(E)** Tumor formation in subcutaneous tumors of the sh-NC and sh-*TMEM105* Mia PaCa-2 cells. **(F)** Tumor growth curves and the weight comparation of the *TMEM105* knockdown group and the control group in Mia PaCa-2 cells. **(G)** Ki67 staining and Tunel assays of the *TMEM105* knockdown group and the control group in Mia PaCa-2 cells. **(H)** The expression of *TMEM105* in *TMEM105* knockdown group and the control group in Mia PaCa-2 cells. *p < 0.05, **p < 0.01, ***p < 0.001, ****p < 0.0001.

In order to verify the effect of *TMEM105* on the proliferation and migration ability of PCa cells, CCK8, colony formation, EdU detection and transwell assays were performed. Mia CaPa-2 and SW1990, which expressed higher level of *TMEM105*, were selected to perform RNA interference (Figure S8-9). As a result, knockdown of *TMEM105* significantly impaired cells viability and proliferation compared to control group (Figure 3A-B, D). Besides, the transwell assays revealed that knockdown of *TMEM105* inhibited the cell migration (Figure 3C). Then, *TMEM105* was overexpressed in PANC-1 and Patu8988 (Figure S10). As was expected, cell proliferation and migration were enhanced by *TMEM105* overexpression (Figure S11-13). To investigate the impact of *TMEM105* in PCa *in vivo*, shRNA lentiviruses were constructed to infect Mia PaCa-2 cells and stably downregulate the expression of *TMEM105*. Cells were then subcutaneously implanted into posterior axillary line of nude mice. After four weeks, the transplanted tumors were excised, and RNA was extracted from tumor tissue for knockdown validation efficiency (Figure 3H). It was revealed there was a significant reduction in term of both volume and weight of the tumor components following *TMEM105* downregulation compared to the control group (Figure 3F). In addition, Ki-67 staining and TUNEL staining confirmed that *TMEM105* knockdown inhibited pancreatic cancer cell proliferation and induced cell apoptosis *in vivo* (Figure 3G). Finally, in order to further invalidate the effect of *TMEM105* on PCa progression *in vivo*, PDX models were constructed. *TMEM105* were found stimulate the progression of PCa under the circumstance of PDX models (Figure S14). In conclusion, *TMEM105* promotes the PCa progression both *in vitro* and *in vivo*.

### TMEM105 mitigates disulfidptosis induced by glucose deprivation in PCa

**Figure 4:**
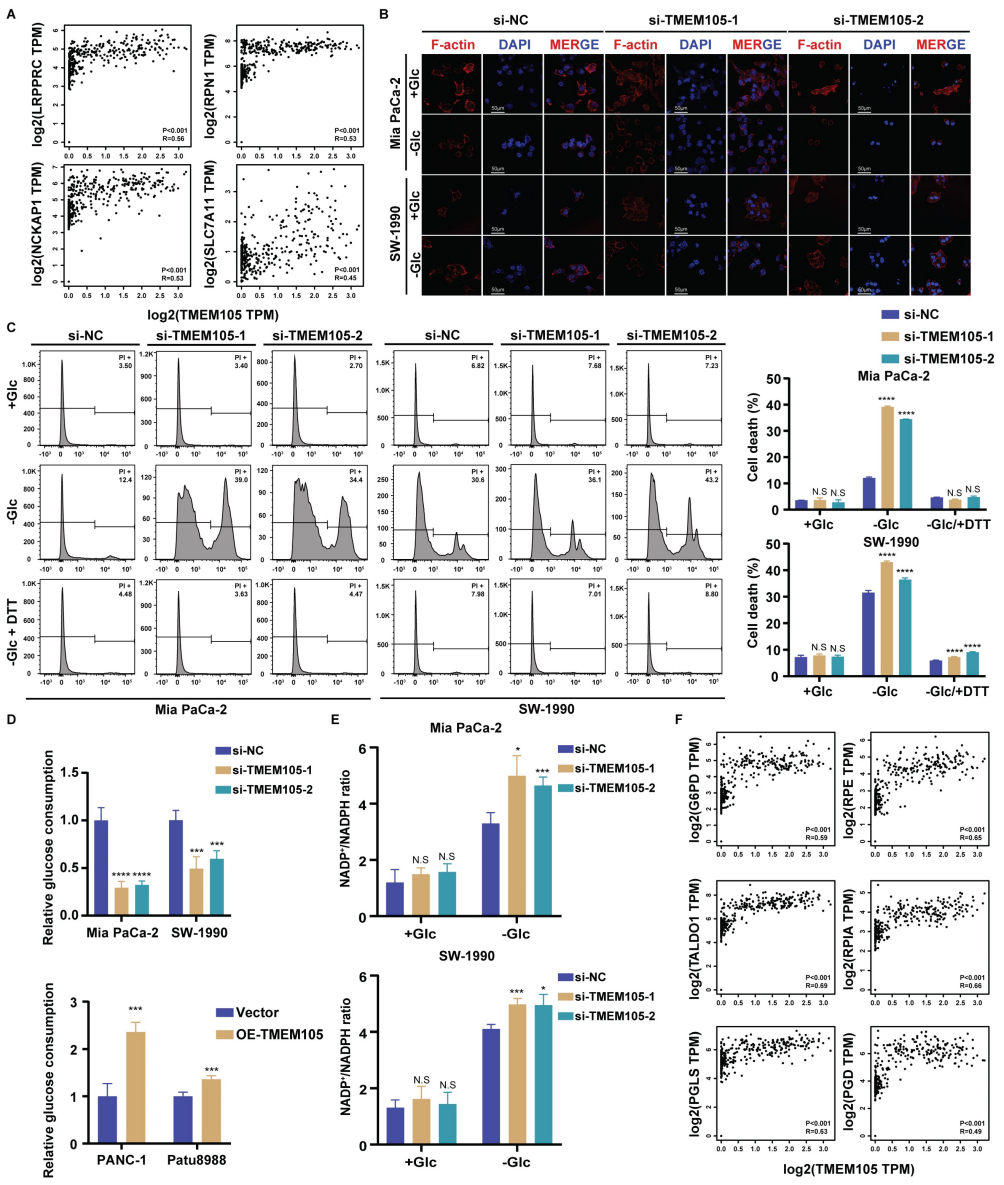
***TMEM105* mitigates the PCa disulfidptosis. (A)** The correlation between *TMEM105* and disulfidptosis-related genes analyzed by GEPIA website based on TCGA and GTEx database. **(B)** F-actin staining of *TMEM105* knockdown Mia PaCa-2 and SW-1990 cells maintained in glucose-free medium for 12 h (scale bar: 50 μm) **(C)** The *TMEM105* knockdown Mia PaCa-2, SW-1990 cells were maintained in glucose-free medium with 0.25 mM DTT for 12 h and subjected to cell death staining. **(D)** The glucose consumption ability of the cells was evaluated in *TMEM105* knockdown Mia PaCa-2, SW-1990 cells or *TMEM105*-overexpressing PANC-1, Patu8988 cells. **(E)** The *TMEM105*-knocking-down Mia PaCa-2 and SW-1990 cells were maintained in glucose-free medium for 12 h and subjected to NADP^+^/ NADPH detection. **(F)** The correlation between *TMEM105* and PPP key genes analyzed by GEPIA website based on TCGA and GTEx database. *p < 0.05, **p < 0.01, ***p < 0.001, ****p < 0.0001.

To verify whether *TMEM105* was associated with disulfidptosis in PCa, we firstly analyzed the correlation between *TMEM105* and disulfidptosis-related genes by GEPIA website based on TCGA and Genotype Tissue Expression (GTEx) database. The results demonstrated a strong correlation (Figure 4A and S15). Then PI staining was applied to detect the cell death. As was expected, there was no significant difference in term of cell death between si-*TMEM105* and si-NC cells when cultured in glucose-containing medium. However, in glucose-free environment, knockdown of *TMEM105* significantly augmented the cell death while *TMEM105* overexpression restored the death rate. Furthermore, the application of disulfidptosis inhibitor DTT could also inhibit the death caused by glucose deprivation, confirming that this type of cell death is caused by *TMEM105* mitigated disulfidptosis (Figure 4C and S16). Based on previous studies, inhibiting GLUT1 reduces cellular glucose uptake, thus leads to dysregulation of PPP and imbalance of NADP^+^/NADPH, resulting in collapse of the cell skeleton and ultimately disulfidptosis 9. To further confirm that *TMEM105* mitigated the cell death by disulfidptosis, cytoskeleton staining and NADP^+^/NADPH detection assays were conducted. As a result, the *TMEM105* knockdown group exhibited more pronounced F-actin contraction and cell shrinkage in the glucose-free environment (Figure 4B). Subsequently, we detected the intracellular cellular glucose uptake and NADP^+^/NADPH ratio. It was found that in *TMEM105* knockdown cells, the intracellular glucose levels decreased significantly (Figure 4D). In addition, downregulation of *TMEM105* increased intracellular NADP^+^/NADPH ratio greatly in the glucose-free environment (Figure 4E). In consist with our expectation, the opposite results were observed in the *TMEM105* overexpressed cells (Figure 4D and S17-18). Finally, the correlation analysis (GEPIA website based on TCGA and GTEx database) explained the mechanism of disulfidptosis -related PPP dysregulation (key genes in PPP was attained from previous study 23) (Figure 4F and S19). In conclusion, *TMEM105* regulates the cell skeleton, glucose uptake, ratio of NADP^+^/NADPH to ultimately mitigate disulfidptosis.

### GLUT1 is essential for the oncogenic roles of TMEM105 in PCa growth and disulfidptosis

**Figure 5:**
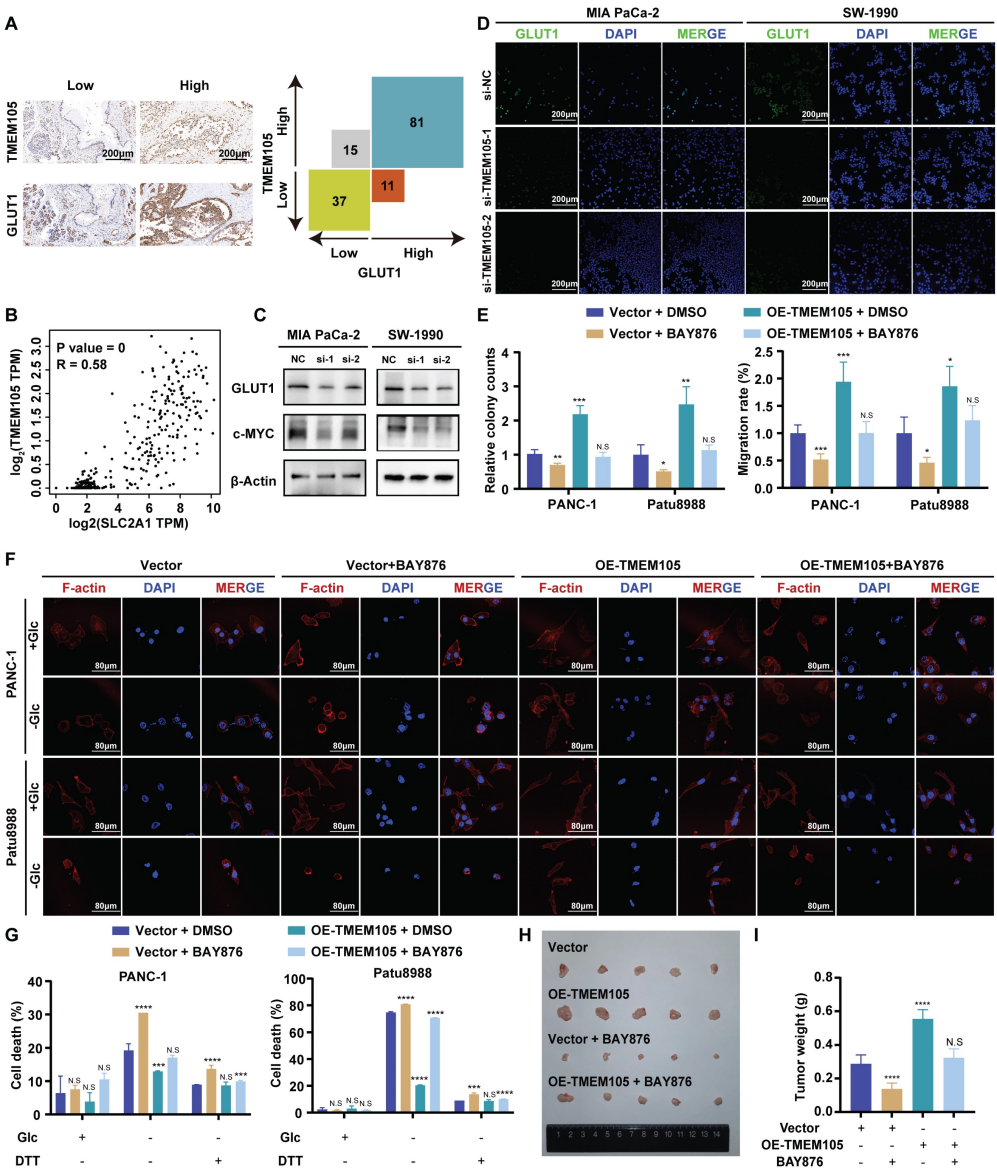
***GLUT1* is essential for the oncogenic roles of *TMEM105* in PCa growth and disulfidptosis. (A)** Staining and analyzing the correlation between *TMEM105* and GLUT1 in consecutive sections of PCa tumor slide from Renji cohort (scale bar: 200 μm). **(B)** Correlation of *TMEM105* with *GLUT1* analyzed by GEPIA website based on TCGA and GTEx database. **(C)** WB analysis of GLUT1 and c-MYC in *TMEM105* knockdown Mia-PaCa 2 and SW-1990 cells (si-1: si-TMEM105-1, si-2: si-TMEM105-2). **(D)** Fluorescence staining of GLUT1 expression in *TMEM105* knockdown cells (scale bar: 200 μm). **(E)** Colony formation assay and transwell assays of *TMEM105*-overexpressing PANC-1 and Patu8988 cells treated with DMSO or 5 µM BAY-876 for 6 hours (scale bar: 1 cm, scale bar: 100 μm). **(F)** F-actin staining of *TMEM105*-overexpressing PANC-1 and Patu8988 cells treated with DMSO or 5µM BAY-876 for 6 h, followed by glucose-deprivation for 12 h (scale bar: 80 μm). **(G)** Cell death staining of *TMEM105*-overexpressing PANC-1 and Patu8988 cells treated with DMSO or 5µM BAY-876 for 6 h, followed by glucose-deprivation for 12 h. **(H)** Representative images of subcutaneous tumors of the *TMEM105*-overexpressing group and the control group after receiving BAY-876 treatment (BAY-876 3mg/kg, oral administration once a week for a total of 4 treatments). **(I)** The weight of subcutaneous tumors mentioned above. *p < 0.05, **p < 0.01, ***p < 0.001, ****p < 0.0001.

Glucose transporter proteins (GLUT) constitute a protein family which is responsible for glucose transport. This family contains fourteen members including *GLUT1* encoded by the *SLC2A1* gene, which plays a significant role in the development of cancer. *GLUT1* is highly expressed in various malignant tumors. Notably, its elevated expression in pancreatic cancer predicts a poor prognosis. *GLUT1* is located in the cell membrane to facilitate glucose's entry into cells. Its biological function closely correlates with glucose metabolism, particularly glycolysis 24-27. Previous studies have linked the occurrence of disulfidptosis to the accumulation of disulfides. This process derived from the reduction of NADP^+^/NADPH caused by inhibiting *GLUT1* 9*.* Thus, we speculated that *TMEM105* may regulate the tumor progression and disulfidptosis in PCa through *GLUT1*. To verify this, 149 pancreatic cancer tissue samples, in consecutive sections, were subjected to IHC and ISH staining for detection of the *TMEM105* and GLUT1 expression. Correlation analysis revealed a positive association between the expressions of *TMEM105* and GLUT1 (Figure 5A). This positive correlation between *TMEM105* and *GLUT1* was also confirmed by GEPIA website (Figure 5B). In addition to that, the association was verified at GLUT1 protein level using WB and IF staining (Figure 5C-D). Besides, it was found that overexpression of *TMEM105* resulted in increased proliferation and migration abilities of PANC-1 and Patu8988 cells, which were attenuated upon the addition of the GLUT1 inhibitor BAY-876 (Figure 5E and S20-21). Moreover, in the glucose-free environment, BAY-876 reversed the inhibited disulfidptosis caused by *TMEM105* overexpression (Figure 5F-G).

To further investigate the impact of *TMEM105* on PCa progression *in vivo*, stable overexpressing Patu8988 cell lines were established and subcutaneously implanted into the posterior axillary line of nude mice, administered orally with or without BAY-876. After four weeks, the tumors were extracted. Intriguingly, oral administration of BAY-876 restored the augmentation of tumor weight and volume caused by *TMEM105* overexpression (Figure 5H-I and S21). Additionally, Ki-67 staining and TUNEL assays respectively indicated that *TMEM105* overexpression promoted tumor proliferation and inhibited tumor apoptosis, while inhibition of GLUT1 reversed this effect (Figure S22). In a word, *GLUT1* is essential for the oncogenic roles of *TMEM105* in PCa growth and disulfidptosis.

### TMEM105 engages the glycolysis-related transcription factor c-MYC to induce GLUT1

**Figure 6:**
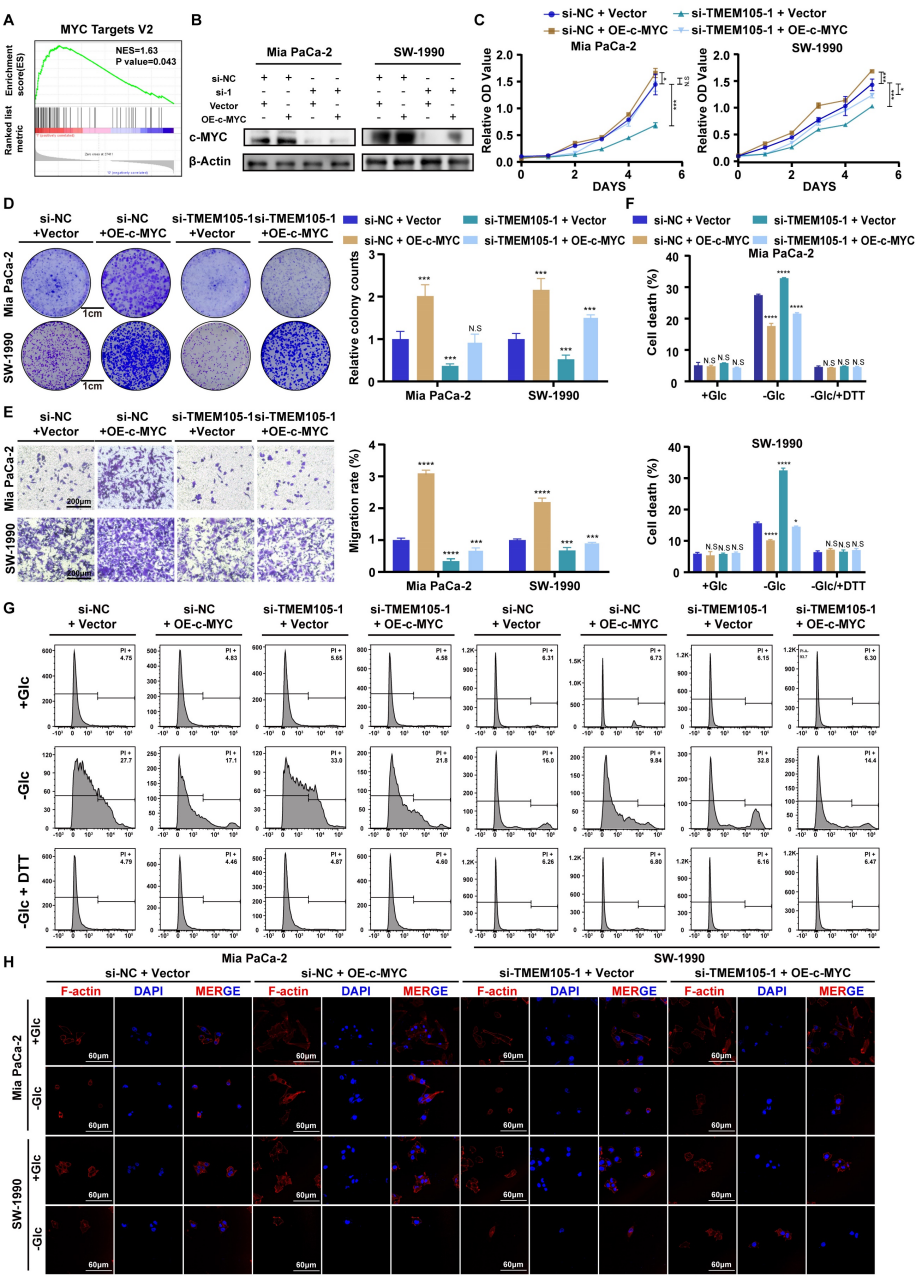
***TMEM105* engages the glycolysis-related transcription factor c-Myc to induce GLUT1**. **(A)** GSEA enrichment analysis of *TMEM105* based on the TCGA database. **(B)** WB analysis of c-MYC in *TMEM105*-knockdown-*c-MYC*-overexpressing Mia PaCa-2 and SW-1990 cells (si-1: si-*TMEM105*-1). **(C)** The cell viability assay of *TMEM105*-knockdown-*c-MYC*-overexpressing Mia PaCa-2 and SW-1990 cells. **(D)** The colony formation assays of *TMEM105*-knockdown-*c-MYC*-overexpressing Mia PaCa-2 and SW-1990 cells (scale bar: 1 cm). **(E)** The transwell assays of *TMEM105*-knockdown-*c-MYC*-overexpressing Mia PaCa-2 and SW-1990 cells (scale bar: 100 μm). **(F, G)** Cell death staining of *TMEM105*-knockdown-*c-Myc*-overexpressing Mia PaCa-2 and SW-1990 cells maintained in glucose-free medium for 12h. **(H)** The F-actin staining of *TMEM105*-knockdown-*c-Myc*-overexpressing Mia PaCa-2 and SW-1990 cells maintained in glucose-free medium for 12h (scale bar: 40 μm). *p < 0.05, **p < 0.01, ***p < 0.001, ****p < 0.0001.

To further explore the specific regulatory mechanism of *TMEM105* regulating GLUT1 in PCa growth and disulfidptosis, GSEA analysis based on TCGA database was performed 28, 29. Interestingly, we noticed that *TMEM105* was significantly associated with the c-MYC pathway (Figure 6A). It is noteworthy that c-MYC is a glycolysis-related transcription factor which plays a vital role in transcriptionally regulating GLUT1 30, 31. Subsequently, to validate our speculation that the essential role of *TMEM105* on GLUT1 is dependent on the glycolysis-related transcription factor c-MYC, cell lines with *TMEM105* interference were established followed by overexpression of c-MYC (Figure 6B). As a result, the decreased proliferation (Figure 6C-D) and migration (Figure 6E) abilities of Mia CaPa-2 and SW-1990 cells brought by *TMEM105* knockdown could be reversed by c-MYC overexpression. Furthermore, overexpression of c-MYC was found to inhibit disulfidptosis due to reduced *TMEM105* levels in term of cell death (Figure 6F-G) and cell cytoskeleton (Figure 6H).

In conclusion, *TMEM105* engages the glycolysis-related transcription factor c-Myc to induce GLUT1.

### TMEM105 maintains the stability of β-catenin to enhance c-MYC expression

**Figure 7:**
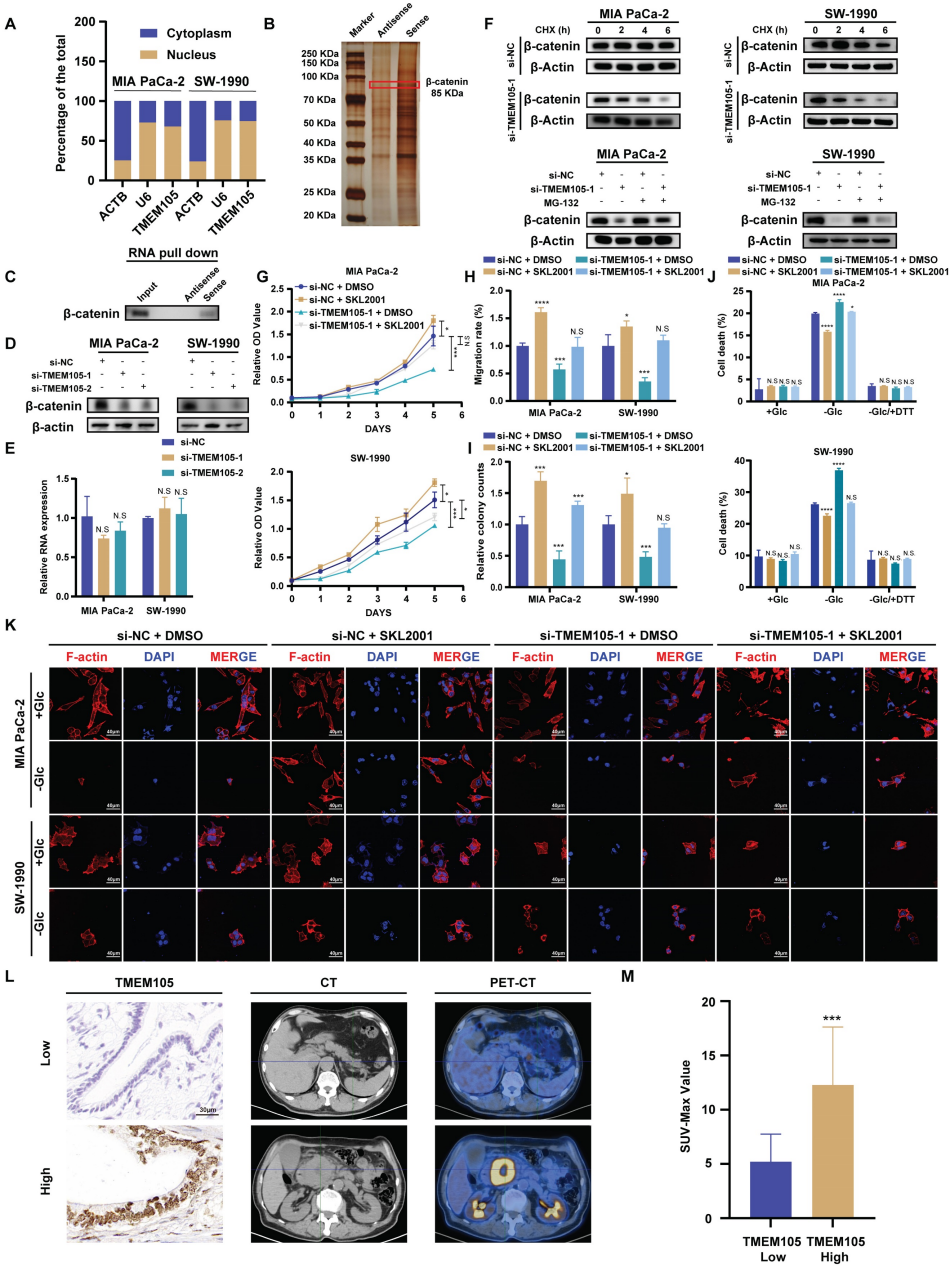
** Reduced *TMEM105* inhibited tumor progression and enhanced disulfidptosis by β-catenin in pancreatic cancer. (A)** qRT-PCR analysis of the subcellular distribution of *TMEM105*, with U6 as a nuclear marker and ACTB as a cytoplasmic marker. **(B)** Protein silver staining image after biotin-labeled RNA pulldown experiment. **(C)** RNA-pulldown assays to validate the interaction between *TMEM105* and β-catenin. **(D)** WB analysis of β-catenin in *TMEM105*-knocking-down cells. **(E)** qRT-PCR of *β-catenin* in *TMEM105* knockdown Mia PaCa-2 and SW-1990 cells at the RNA level. **(F)** WB analysis of *TMEM105* knockdown Mia PaCa-2 and SW-1990 cells treated with 20 μg/mL CHX at indicated time or 10 μM MG132 for 6 h. **(G)** The cell viability assay of *TMEM105*-knockdown-SKL2001-treated Mia PaCa-2 and SW-1990 cells. **(H)** The colony formation assays of *TMEM105*-knockdown-SKL2001-treated Mia PaCa-2 and SW-1990 cells. **(I)** The transwell assays of *TMEM105*-knockdown-SKL2001-treated Mia PaCa-2 and SW-1990 cells. **(J)** Cell death staining of *TMEM105*-knockdown-SKL2001-treated Mia PaCa-2 and SW-1990 cells maintained in glucose-free medium for 12h. **(K)** F-actin staining of *TMEM105*-knockdown-SKL2001-treated Mia PaCa-2 and SW-1990 cells maintained in glucose-free medium for 12h (scale bar: 40 μm). **(L)** Representative ISH, CT, and PET-CT images of *TMEM105* low- and high-expression groups in 14 patients with pancreatic cancer from Renji Hospital, the relationship between *TMEM105* expression and SUV-Max was analyzed. **(M)** Statistical analysis of SUV-max values in 14 patients with pancreatic cancer from Renji Hospital. *p < 0.05, **p < 0.01, ***p < 0.001, ****p < 0.0001.

To further explore the mechanism, the cell cytoplasm and nucleus were separated. It was observed that *TMEM10*5 (Figure 7A) located in the nucleus, which was consistant with the results of ISH staining (Figure 2C). Then, RNA pull-down was employed and the distinct protein bands were analyzed through LC-MS/MS. However, the interaction between *TMEM105* and c-MYC was not observed, which indicated that the regulation of c-MYC by *TMEM105* is indirect. Thus, we screened the proteins interacted with *TMEM105* and finally narrowed down to β-catenin, which was reported to interacted with c-MYC directly (Figure 7B) 32-34. β-catenin, a key intracellular signaling protein expressed in various cell compartments, plays a fundamental role in regulating cell proliferation, differentiation, and migration 35, 36. It was reported that aberrant activation of β-catenin is closely associated with pancreatic cancer progression (Figure S23) 37.

Notably, β-catenin-c-MYC signaling pathway is crucial in pancreatic cancer metabolism, especially in the regulation of glucose metabolism 38-40. To verify this, WB analysis was performed after incubating biotin-labeled *TMEM105* sense and antisense RNA products and confirmed the direct interaction of *TMEM105* and β-catenin (Figure 7C). LncRNAs are commonly reported to regulate the stability of binding proteins 41-43. Hence, we hypothesized that *TMEM105* may exert its regulatory effects on pancreatic cancer progression and disulfidptosis through interacting with β-catenin. Importantly, it was noticed that the protein levels of β-catenin significantly decreased after *TMEM105* knocking down (Figure 7D), while the mRNA levels of *β-catenin* remained unaffected (Figure 7E). The results above suggested that the regulatory role of *TMEM105* on β-catenin occurred at the translational or post-translational level. Cycloheximide (CHX) is frequently used to assess protein half-life and stability 44. Therefore, to validate the assumption of *TMEM105* regulating β-catenin protein stability, CHX was added to Mia CaPa-2 and SW-1990 cells for WB analysis. It was showed a significant reduction in the half-life of β-catenin after downregulation of *TMEM105*. Subsequently, to elucidate how *TMEM105* maintains β-catenin protein stability, proteasome inhibitor MG132 was introduced to Mia CaPa-2 and SW-1990 cells. The inhibitor eliminated the alteration of β-catenin expression caused by the loss of *TMEM105* functionality (Figure 7F). Collectively, these findings suggest that *TMEM105* maintains the protein stability of β-catenin by the proteasome pathway. Additionally, GEPIA analysis based on TCGA database verified the correlation between *β-catenin* and *TMEM105.* Furthermore, the upregulated β-catenin increased the expression of c-MYC (Figure S24-25). In conclusion, *TMEM105* maintains the stability of β-catenin by the proteasome pathway to enhance c-MYC expression.

### TMEM105 interacts with β-catenin in PCa

To further demonstrate the role of *TMEM105* in regulating pancreatic cancer growth and disulfidptosis through β-catenin, Mia PaCa-2 and SW-1990 were treated with β-catenin activator SKL2001 (20μM) after *TMEM105* knockdown. Through CCK-8 and colony formation assays, it was indicated that SKL2001 restored the suppressed proliferative capacity caused by *TMEM105* knockdown (Figure 7G-H and S26). Besides, the cell migration capability of *TMEM105* interfered cell lines was also enhanced with the treatment of SKL2001 through transwell assays (Figure 7I and S27). These findings suggested that *TMEM105* stimulated the proliferation and metastasis of pancreatic cancer via β-catenin pathway. Moreover, SKL2001 was found to reverse the contraction of F-actin and the shrinkage of the cell cytoskeleton in the glucose-free environment induced by *TMEM105* knockdown (Figure 7K). More importantly, SKL2001 also reduced cell death in the glucose-free environment resulting from downregulation of *TMEM105* (Figure 7J). Take it to conclude, *TMEM105* mitigates the pancreatic cancer disulfidptosis through combining with β-catenin.

In conclusion,* TMEM105* regulates pancreatic cancer growth and disulfidptosis through β-catenin.

### Validation of TMEM105-β-catenin-c-MYC-GLUT1 pathway in clinical settings

2-Fluoro-18-fluoro-2-deoxy-D-glucose (18F-FDG) is an analogue of glucose. After intravenous injection of 18F-FDG, it can be transported into cells via glucose transporters. Compared to normal tissues, tumors exhibit increased glucose metabolism. Therefore, in clinical, 18F-FDG is commonly used as a tracer in PET-CT scans to assess tumor progression 45-47. To further validate that *TMEM105* regulated pancreatic cancer growth and disulfidptosis through the β-catenin-c-MYC-GLUT1 pathway, tissue samples from pancreatic cancer patients with PET-CT information were utilized. The samples in consecutive sections were subjected to ISH and IHC staining to examine the correlation between the *TMEM105*-β-catenin-c-MYC-GLUT1 axis and glucose metabolism. As was shown, the expression of *TMEM105* was positively correlated with the standard uptake value (SUV-Max) on the corresponding PET-CT scans of the patients (Figure 7L). Additionally, in cases with high *TMEM105* expression, both c-MYC and GLUT1 were also highly expressed (Figure S28). In summary, clinical samples from PCa patients containing PET-CT information further confirm the role of *TMEM105* in regulating PCa progression and disulfidptosis through the β-catenin-c-MYC-GLUT1 pathway.

## Discussion

PCa is one of the most malignant tumors globally. In recent years, despite some progress in screening and treatment of PCa, it is still challenging to accurately identify and screen high-risk populations. The effectiveness of treatments such as surgery and chemotherapy are also not satisfactory. Hence, identifying biomarkers to predict PCa prognosis in conjunction with targeted therapy presents as a problem to be solved. Different from apoptosis and ferroptosis, disulfidptosis is a novel type of cell death and cannot be suppressed by conventional cell death inhibitors. The characteristic of disulfidptosis includes the massive uptake of cysteine, glucose starvation, and depletion of NADPH, with the most important being the accumulation of intracellular disulfide bond molecules. In glucose-deprived cancer cells, the high uptake of cysteine and inadequate NADPH supply jointly contribute to abnormal accumulation of disulfide bond compounds, which bind with actin cytoskeletal proteins, ultimately causing the collapse of the actin cytoskeleton network and cell death. Therefore, investigating disulfidptosis in PCa can provide further insights into the molecular mechanisms during development of cancer. Undoubtedly, targeting this unique form of cell death may become a potential molecular marker for screening and treating PCa.

In this study, we utilized bioinformatics analysis to develop a risk signature consisting of six lncRNAs associated with disulfidptosis for predicting the prognosis of PCa. Through analysis of expression and prognosis significance of PCa by GEPIA database and PCa samples from Renji Hospital, we identified the critical gene *TMEM105*. *TMEM105* was a lncRNA that plays a role in promoting breast cancer growth and metastasis by regulating tumor glycolysis function in breast cancer liver metastasis 13. It served as a cell cycle-related lncRNA for predicting progression-free survival time in thyroid papillary carcinoma,^14^ and as an ferroptosis- and immune-related lncRNA for predicting patient prognosis in breast infiltrating duct and lobular carcinoma 15. *TMEM105* was also believed to play a crucial role in coronary artery disease 16. In this study, *TMEM105* was found to promote pancreatic cancer progression *in vivo* and *in vitro*, and mitigate disulfidptosis of PCa *in vitro*. However, its exact function in PCa has not been unveiled.

Targeting *GLUT1* has been shown to significantly inhibit pancreatic cancer progression 25, 48. Previous research has linked the occurrence of disulfidptosis to the increase of NADP^+^/NADPH resulting from *GLUT1* inhibition,^9^ which was also confirmed in this study. In our research, GSEA enrichment analysis was performed to find out the notable association of *TMEM105* with c-MYC pathway. It was found that there was a significant positive correlation between c-MYC expression and *TMEM105*. In addition, the impact of *TMEM105* on GLUT1 was dependent on c-MYC through interacting with β-catenin. As is reported, β-catenin-c-MYC signaling pathway participates in regulating various cellular functions in tumors. For example, in colorectal cancer, *CD36* promoted GPC4 ubiquitination via proteasome-dependent pathway and exerts inhibitory effects on glycolysis through the β-catenin-c-MYC signaling pathway 49. *MUC5AC* intensified glutamine utilization and nucleotide biosynthesis through the β-catenin-c-MYC pathway, thus enhancing pancreatic cancer resistance to gemcitabine 38. Combined chemotherapy with niclosamide and gemcitabine induced β-catenin ubiquitination, consequently downregulated the β-catenin-c-MYC signaling pathway to inhibit pancreatic cancer progression 39. In this experiment, stimulating β-catenin in *TMEM105*-knockdown cells led to the suppression of disulfidptosis and the restoration of proliferation and metastatic capabilities of pancreatic cancer cells. However, this study also has some limitations. Firstly, a logical *in vivo* validation model for disulfidptosis was not provided and established. Secondly, the specific regulatory mechanism of *TMEM10*5 on the downstream β-catenin-c-MYC-GLUT1 signaling axis needs further elucidation.

In summary, this study reveals that *TMEM105* is highly expressed in PCa and indicates poor prognosis. It exerts its regulatory effects on the progression of pancreatic cancer and disulfidptosis through the β-catenin-c-MYC-GLUT1 signaling axis. This study has confirmed *TMEM105* mitigates disulfidptosis in PCa for the first time, and has elucidated the potential mechanism in regulating disulfidptosis.

## Supplementary Material

Supplementary figures.
