# Supplementary material for: TMEM105 modulates disulfidptosis and tumor growth in pancreatic cancer via the β-catenin-c-MYC-GLUT1 axis
Source: Int J Biol Sci. 2025 Feb 18;21(5):1932–48. doi: 10.7150/ijbs.104598 (PMC11900826; doi:10.7150/ijbs.104598)
Supplement: Supplementary file 1 — Supplementary figures. [file ijbsv21p1932s1.pdf]

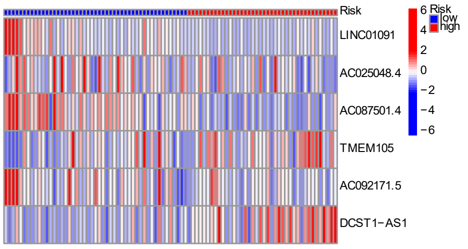

Figure S1. Risk heatmap of the testing set

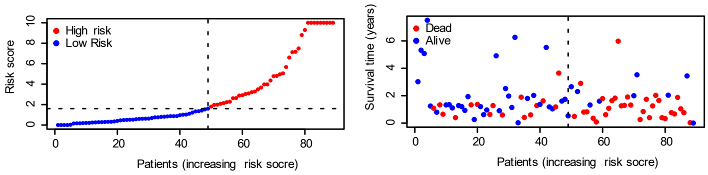

Figure S2. Distribution plot of risk scores and scatter plot of survival status in the training set.

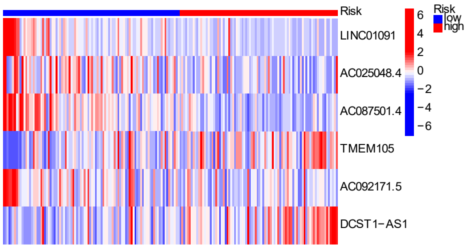

Figure S3. Risk heatmap of the entire set

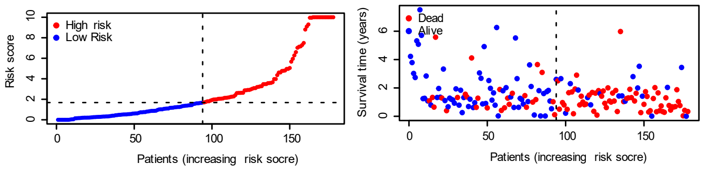

Figure S4. Distribution plot of risk scores and scatter plot of survival status in the entire set.

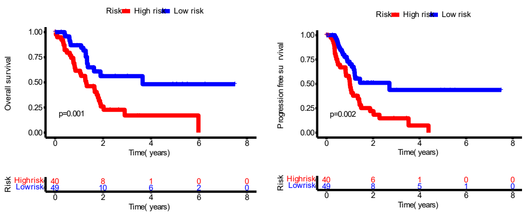

Figure S5. KM analysis for OS and PFS in the testing set based on the TCGA database

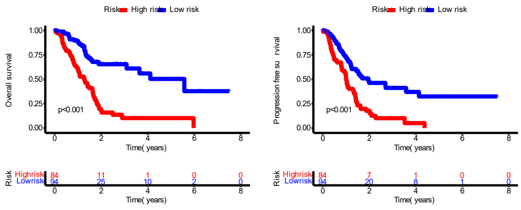

Figure S6. KM analysis for OS and PFS in the entire set based on the TCGA database

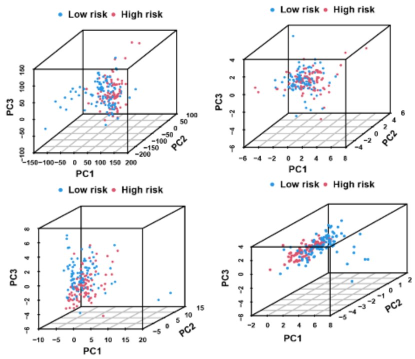

Figure S7. PCA plot of all genes, disulfidptosis-related genes, disulfidptosis-related lncRNAs and risk signature.

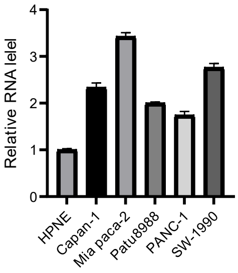

Figure S8. The RNA level of *TMEM105* in HPNE and five pancreatic cancer cell lines.

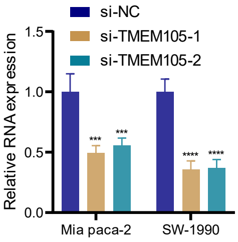

Figure S9. The RNA level of *TMEM105* in *TMEM105* knockdown Mia CaPa-2 and SW1990.

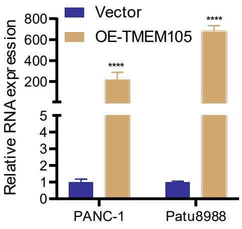

Figure S10. The RNA level of *TMEM105* in *TMEM105*-overexpressing PANC-1 and Patu8988.

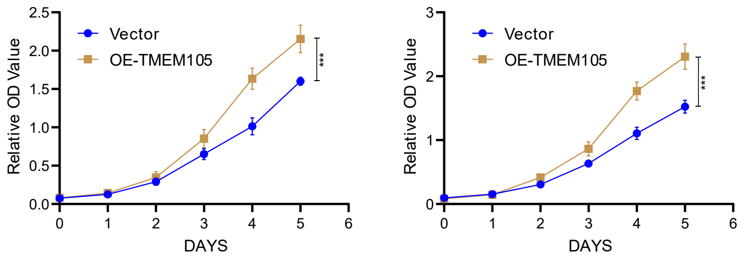

Figure S11. Cell viability was detected in *TMEM105*-overexpressing PANC-1 and Patu8988 cells.

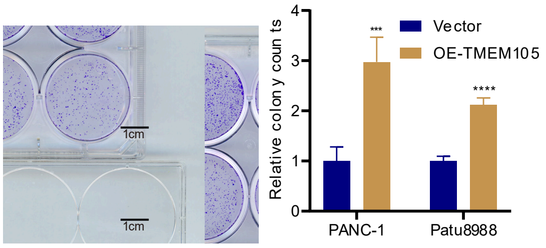

Figure S12. The colony formation assays were conducted in *TMEM105*-overexpressing PANC-1 and Patu8988 cells (scale bar: 1 cm).

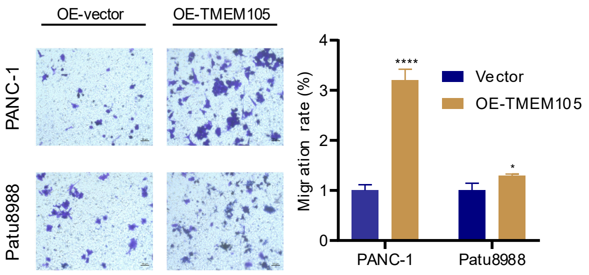

Figure S13. Cell migration was detected in *TMEM105*-overexpressing PANC-1 and Patu8988 cells (scale bar: 50 μm).

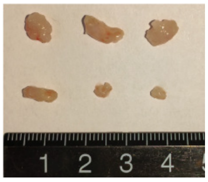

Figure S14. Representative images of PDX tissues

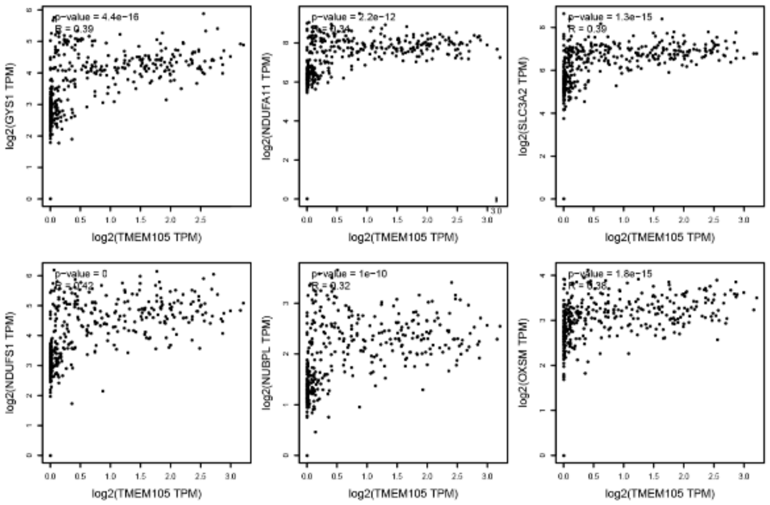

Figure S15. The correlation between *TMEM105* and disulfidptosis-related genes analyzed by GEPIA website based on TCGA and GTEx database.

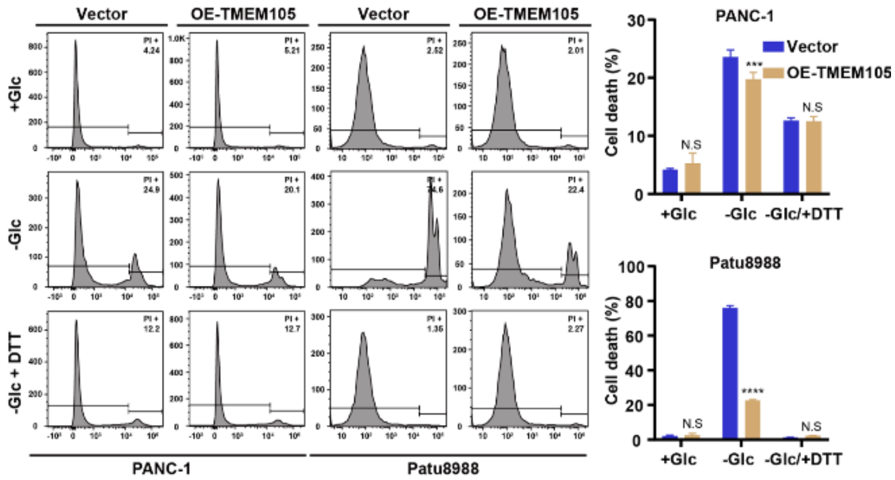

Figure S16. The *TMEM105*-overexpressing PANC-1 and Patu8988 cells were maintained in glucose-free medium with 0.25 mM DTT for 12 h and subjected to cell death staining.

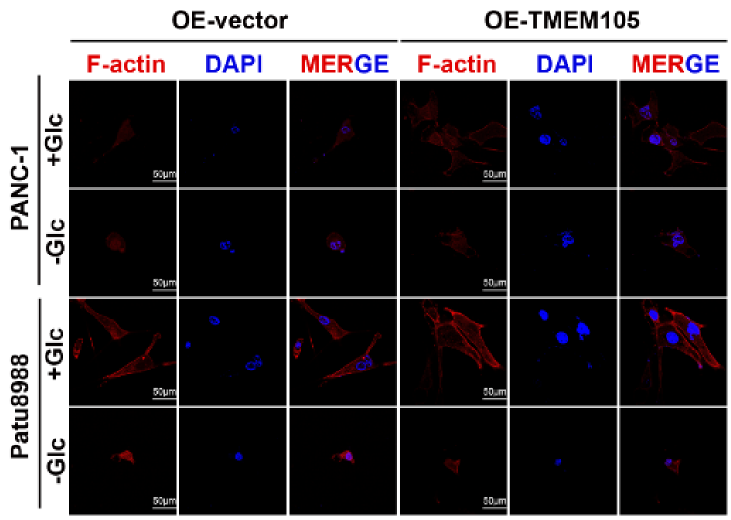

Figure S17. F-actin staining of *TMEM105*-overexpressing PANC-1 and Patu8988 cells maintained in glucose-free medium for 12 h (scale bar: 50 μm)

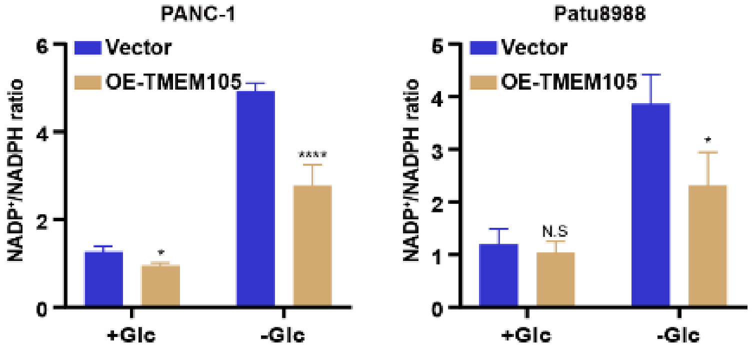

Figure S18. The *TMEM105*-overexpressing PANC-1 and Patu8988 cells were maintained in glucose-free medium for 12 h and subjected to $NADP^+$/ NADPH detection.

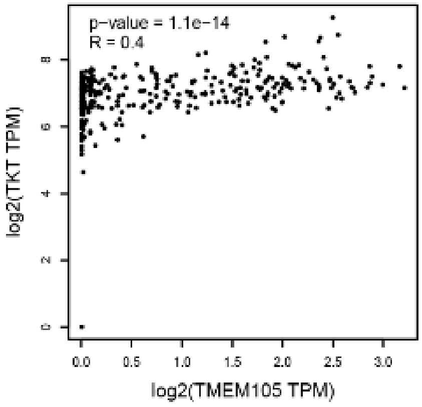

Figure S19. The correlation between *TMEM105* and *TKT* (one of PPP key genes) analyzed by GEPIA website based on TCGA and GTEx database.

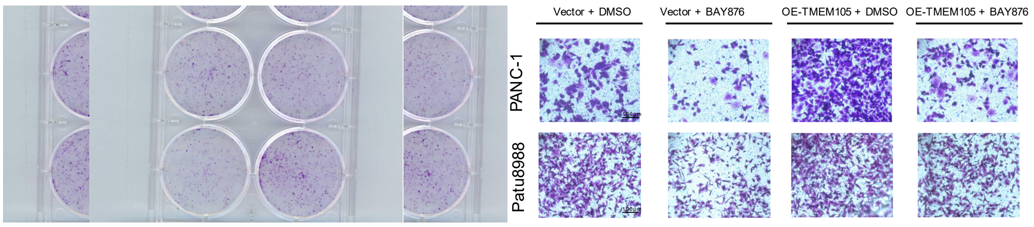

Figure S20. The colony formation and the transwell assays of *TMEM105*-overexpressing PANC-1 and Patu8988 cells treated with DMSO or 5 μM BAY-876 for 6 hours (scale bar: 1 cm; scale bar: 100 μm)

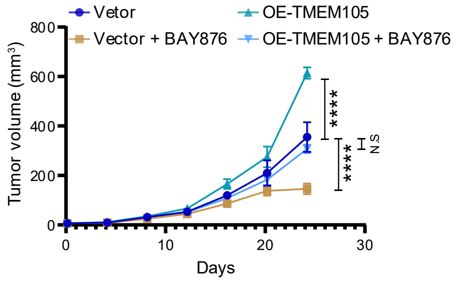

Figure S21. The tumor volume in subcutaneous tumors of the *TMEM105*-overexpressing group and the control group after the treatment mentioned above.

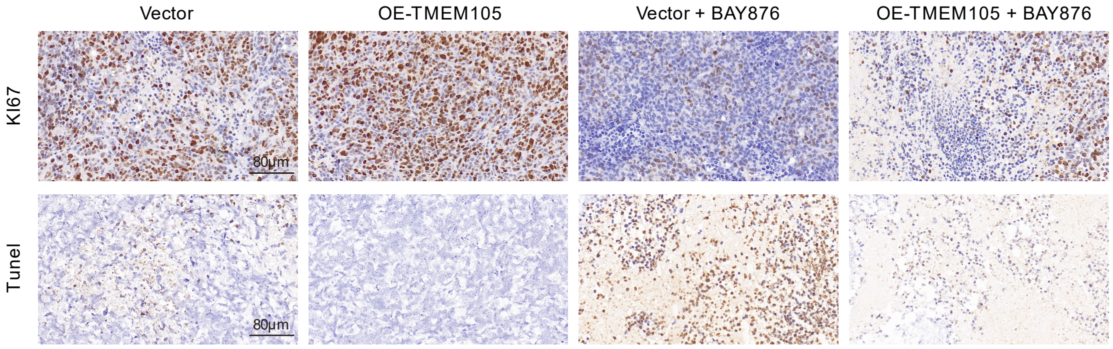

Figure S22. The KI67 staining and Tunel assays after the treatment mentioned above.

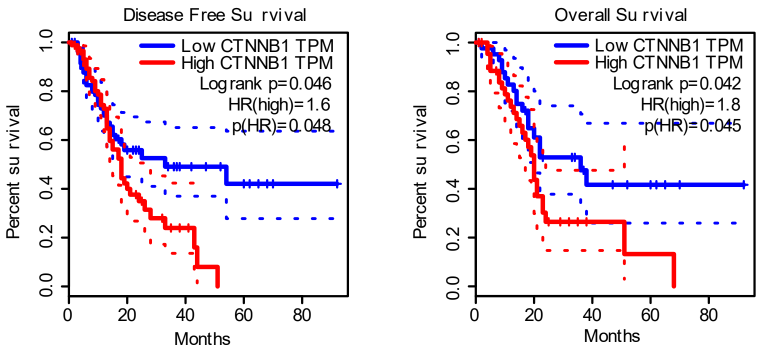

Figure S23. KM analysis for OS and PFS on PCa patients based on the TCGA database

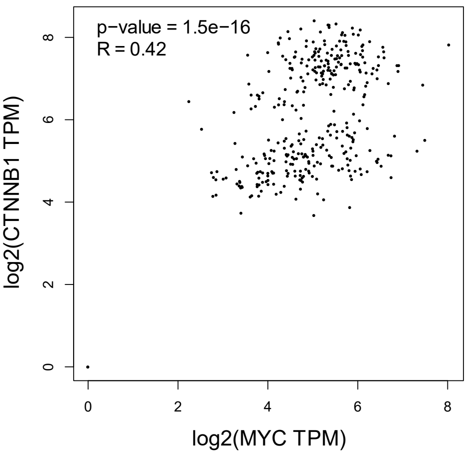

Figure S24. The correlation between *β-catenin* and *c-MYC* analyzed by GEPIA based on TCGA and GTEx database.

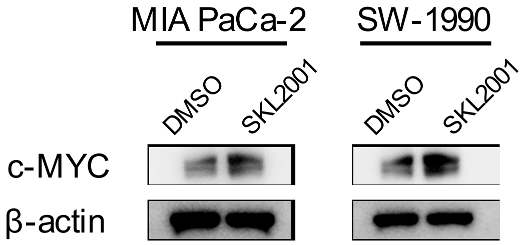

Figure S25. WB analysis of SKL2001-treated Mia PaCa-2 and SW-1990 cells

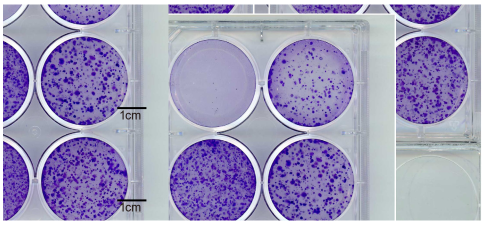

Figure S26. The colony formation assays were performed in *TMEM105*-knockdown-SKL2001-treated Mia PaCa-2 and SW-1990 cells.

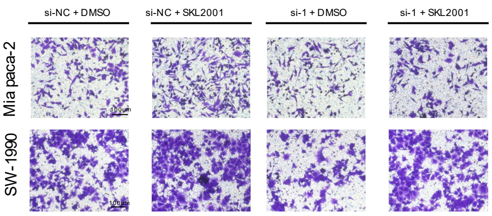

Figure S27. The transwell assays were performed in *TMEM105*-knockdown-SKL2001-treated Mia PaCa-2 and SW-1990 cells (si-1 refers to si-TMEM105-1).

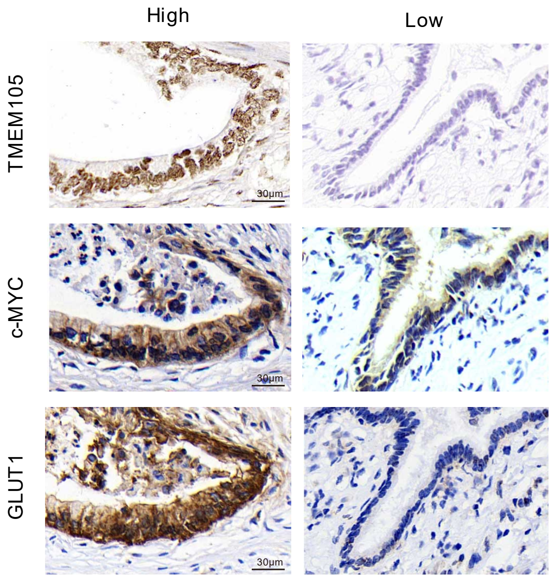

Figure S28. Representative ISH and IHC images of TMEM105, c-MYC and GLUT1 in TMEM105 low- and high-expression groups from 14 pancreatic cancer patients received operations in Renji Hospital
